# Quantification of area-selective deposition on nanometer-scale patterns using Rutherford backscattering spectrometry

**DOI:** 10.1038/s41598-022-22645-8

**Published:** 2022-10-22

**Authors:** Niels Claessens, Zamran Zahoor Khan, Negin Rahnemai Haghighi, Annelies Delabie, André Vantomme, Wilfried Vandervorst, Johan Meersschaut

**Affiliations:** 1 IMEC, Kapeldreef 75, 3001 Leuven, Belgium; 2 Quantum Solid State Physics, KU Leuven, Celestijnenlaan 200D, 3001 Leuven, Belgium; 3 Quantum Chemistry and Physical Chemistry, KU Leuven, Celestijnenlaan 200F, 3001 Leuven, Belgium

**Keywords:** Condensed-matter physics, Nanoscale materials, Techniques and instrumentation, Condensed-matter physics, Techniques and instrumentation

## Abstract

We present a site-specific elemental analysis of nano-scale patterns whereby the data acquisition is based on Rutherford backscattering spectrometry (RBS). The analysis builds on probing a large ensemble of identical nanostructures. This ensures that a very good limit of detection can be achieved. In addition, the analysis exploits the energy loss effects of the backscattered ions within the nanostructures to distinguish signals coming from different locations of the nanostructures. The spectrum deconvolution is based on ion-trajectory calculations. With this approach, we analyse the Ru area-selective deposition on SiO_2_-TiN line-space patterns with a linewidth of 35 nm and a pitch of 90 nm. We quantify the selectivity and the Ru local areal density on the top versus on the sidewall of the SiO_2_ lines. The sensitivity to probe ruthenium deposited on the various surfaces is as low as 10^13^ atoms/cm^2^. The analysis is quantitative, traceable, and highly accurate thanks to the intrinsic capabilities of RBS.

## Introduction

The semiconductor industry continues to develop smaller and better-performing devices. For this, in the last decade the industry has implemented a fast transition from a 2D planar technology to a 3D nano-scale technology^1^. The production of semiconductor devices with nanometer dimensions is enabled by the introduction of advanced processing methodologies such as extreme ultra-violet lithography and area-selective deposition (ASD)^2,3^. Besides, a plenitude of new materials is being introduced to solve the scaling issues^4,5^.

Advanced methods are needed to characterize the composition of such increasingly sophisticated nano-scale patterns to support the further developments in the semiconductor industry^6^. At present, scanning electron microscopy (SEM) and transmission electron microscopy (TEM) in combination with energy dispersive X-ray spectroscopy are intensely pursued to probe the three-dimensional elemental distribution at the nanoscale^7^. However, the strongly reduced analysis volume leads to a lower limit of detection on the order of 0.1 atom % in the bulk or 10^15^ atoms/cm^2^ at the surface or interface. Recently, a new group of techniques has emerged which are based on the principle of the ensemble measurement. This is the simultaneous measurement of a large ensemble of structures and leads to an enhanced sensitivity^8–15^. It is enabled by the excellent uniformity of the nano-scale patterns produced by semiconductor manufacturing^16–18^, characterized by a standard deviation on the size of the nanostructures of the order of 1 nm. However, it has proven to be nontrivial with the existing techniques to distinguish atoms of the same element in different locations on the nanostructures. For example, self-focusing secondary ion mass spectrometry (SIMS) has been shown to provide a good sensitivity on an array of nano-scale structures^8^. Although self-focusing SIMS can discern the same element in a different matrix, the self-focusing SIMS approach does not allow to differentiate between atoms of the same element which are in the same matrix within the different locations on the nanostructures. Very recently, medium energy ion scattering (MEIS) has been demonstrated to differentiate atoms of the same element on the top, sides, and bottom surface of nano-scale structures whereby the smallest quantified local areal density on silicon nanostructures is 2 10^14^ atoms/cm^2^ of arsenic^9,10^.

In the present work, we deploy Rutherford backscattering spectrometry^19^ (RBS) to benefit from its very high sensitivity and its depth resolution, and we demonstrate the analysis of the site-specific area-selective deposition in nano-scale structures. Rutherford backscattering spectrometry is a quantitative characterization method for the atomic areal density, which has been extensively used for its high traceability^20^. Traditionally, the lateral resolving power of Rutherford backscattering spectrometry is improved by focusing the primary ion beam. However, the key issues with focusing the beam to below the sub-micrometre regime are the reduced ion beam current and the concomitant impractical analysis time, and the highly localised fluence causing detrimental beam damage^11,21,22^. Here we instead probe a large ensemble of nanostructures simultaneously, thereby drastically enhancing the sensitivity and avoiding the beam damage. Using this approach, it has been shown that a good sensitivity can be achieved to study the composition of nano-scale structures with RBS^15^. In the present work, we demonstrate that distinguishing atoms of the same element on the top, sides, and bottom surface of nano-scale structures can be achieved by taking advantage of the energy loss, by combining the results from experiments in different geometries, and by analysing the experimental spectra with advanced trajectory simulations. We demonstrate the performance of this approach through the study of the early stages of the area-selective deposition of Ru on SiO_2_-TiN line-space patterns with a linewidth of 35 nm and a pitch of 90 nm^7^.

## Sample preparation and characterization

**Figure 1:**
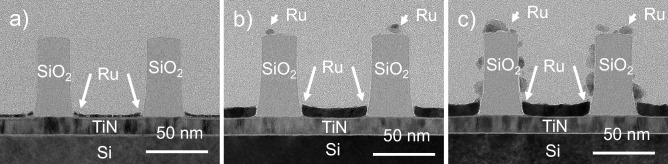
TEM images of (**a**) the Ru/SiO_2_/TiN nanostructures without pre-treatment after 50 s of Ru CVD, (**b**) the DMA-TMS treated Ru/SiO_2_/TiN nanostructures after 300 s of Ru CVD, and (**c**) the Ru/SiO_2_/TiN nanostructures without pre-treatment after 400 s of Ru CVD.

Samples composed of SiO_2_-TiN line-space patterns (Fig. 1) were fabricated on 300 mm diameter Si(100) wafers. A TiN film of 15 nm thickness was deposited by physical vapour deposition followed by the growth of a SiO_2_ film of 75 nm by plasma-enhanced atomic layer deposition (PE-ALD). The mass densities of the TiN and the SiO_2_ compounds are 4.85 (15) g/cm^3^ and 2.00 (10) g/cm^3^, respectively. The photolithographic patterning and etching processes are described elsewhere^8^. Each patterned area on the wafer is 1 cm by 1.2 cm large. The line-space patterns studied in this work have a pitch of 90 nm and a trench width of 55 nm. The SiO_2_ lines are 1 cm long, 35 nm wide, and 75 nm high. Top-view scanning electron microscopy imaging indicates a standard deviation for the linewidth and the pitch below 2 nm across the wafer. The standard deviation for the height and linewidth of the SiO_2_ lines as derived from a TEM analysis is less than 3 nm. We refer the reader to the supplementary material for more details.

The goal of the area-selective deposition is to grow ruthenium only on the TiN surface in-between the SiO_2_ lines while leaving the SiO_2_ lines uncovered. The ruthenium chemical vapour deposition (CVD) was performed using the carbonyl-alkylcyclohexadienyl Ru and hydrogen precursors as described elsewhere^7^. For different samples a deposition time of 50 s, 100 s, 150 s, 200 s, 300 s or 400 s was used. By default, the surface of the SiO_2_ lines is terminated by a hydroxyl (–OH) group^7^. However, one sample was pre-treated using dimethylamino-trimethylsilane (DMA-TMS) to obtain a trimethylsilyl (–Si(CH_3_)_3_) terminated surface on the oxide^23^. The trimethylsilyl termination is believed to enhance the diffusion of Ru adspecies on the surface and is applied to supress the residence of Ru adspecies on the oxide, as such enhancing the selectivity^7^. The DMA-TMS pre-treated sample was treated with 300 s of Ru chemical vapor deposition.

Transmission electron microscopy images were taken with a Tecnai F30 ST microscope operated at 300 kV. The samples were prepared for transmission electron microscopy by first capping them with a thin spin-on carbon layer and then cutting 50-nm-thin lamellae using a focused ion beam Helios450HP instrument.

The transmission electron microscopy images of three selected samples are shown in Fig. 1. The dark colour in between the SiO_2_ lines reveals the presence of Ru at the bottom of the trench. Note that the behaviour on the SiO_2_ lines is qualitatively different for the three samples. The sample without pre-treatment and exposed to 50 s of ruthenium chemical vapor deposition is shown in Fig. 1a. Here, the transmission electron microscopy images did not indicate any ruthenium on the oxide lines, while a ruthenium layer of 3 nm is clearly visible on the TiN area in the trench. The sample which is pre-treated with DMA-TMS and exposed to 300 s of ruthenium chemical vapor deposition, is shown in Fig. 1b. This sample has Ru nanoparticles with an average size of 5 nm on the top of the oxide line, and a Ru layer of 8 nm on the TiN area in the trench. Yet, the sidewalls of the SiO_2_ lines visually appear to be free of Ru. This is in line with the expectations, given the enhanced mobility of Ru adspecies on the oxide mask. The nanostructure without pre-treatment followed by 400 s of Ru chemical vapor deposition is shown in Fig. 1c. This sample features Ru nanoparticles on every surface of the oxide lines with an average size of 7 nm, and a Ru layer of 10 nm on the TiN area.

To quantify the amount of material deposited on the different surfaces with real-space imaging, one would need to know the shape and volume of the clusters and their mass-density as well as the inter-cluster distances. In the case of extremely low coverage, this information cannot be obtained from TEM images. An additional limitation would be the limited statistical information obtained from the analysis of one lamella. Thus, one would need to investigate many lamellae to arrive at the average amount of Ru that is present on the different surfaces. While the presence of Ru nanoparticles can be observed with TEM, it does not readily allow to quantify the amount of Ru on the different surfaces. Therefore, one must investigate the capabilities of alternative approaches.

## Experimental considerations for RBS on nanostructures

The Rutherford backscattering spectrometry experiments were performed using a 1.52 MeV He^+^ beam with a beam current of 15 nA obtained from a tandem 6SDH Pelletron accelerator from National Electrostatics Corporation^24^. The end-station consists of a 5-axis goniometer with the sample tilt and scattering angle in the same plane. The detector is movable and based on a Hamamatsu S3590-09 Si PIN photodiode. The detector solid angle is 1.4 msr. The energy resolution of the set-up is 17.5 keV. The detector was moved to perform experiments in a different geometry.

The beam spot was confined to an area of 2 mm × 2 mm using slits. Thus, more than twenty-two thousand SiO_2_ lines are exposed to the probing beam. Each measurement was recorded with a total applied charge of 60 µC. The sample was moved a few mm in between measurements to minimize beam damage effects^22,25^. With a camera system it was ensured that the area exposed to the probing beam remained well within the same patterned area of 1 cm by 1.2 cm. The pressure remained well below 10^–6^ mbar during the experiments.

**Figure 2:**
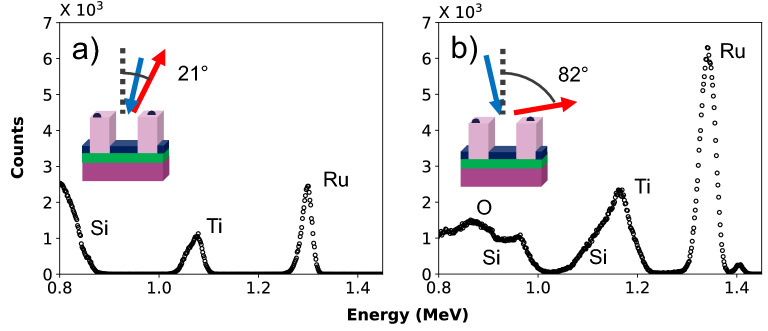
Experimental Rutherford backscattering spectra in (**a**) the geometry with an exit angle of 21° and (**b**) glancing exit geometry of the nanostructures pre-treated with DMA-TMS followed by 300 s of Ru CVD.

First, we want to quantify the total amount of Ru on the sample. In Fig. 2a we show a Rutherford backscattering spectrum obtained on the nanostructures treated with DMA-TMS and followed by 300 s Ru deposition, which is the sample shown in Fig. 1b. The spectrum was obtained with a sample tilt of 11°, a scattering angle of 170° and an exit angle of 21° (see inset of Fig. 2a). The substrate signal was used to determine the solid angle ⋅ charge product^26^. During the data acquisition the sample was rotating along the surface normal to mitigate channeling effects of the ions within the substrate^19^. In the Rutherford backscattering spectrum, the titanium signal appears at 1.1 MeV and the Ru signal at 1.3 MeV. The intensities are a measure for the average areal densities on the sample. We found an average Ru areal density of 27.2 (0.8) 10^15^ atoms/cm^2^ on the sample.

Second, we want to investigate the sensitivity of RBS to the Ru presence on the different sites of the line-space patterns. In Fig. 2b we show the glancing-exit geometry Rutherford backscattering spectrum obtained on the same nano-scale patterns. The sample normal was tilted by 7° off the ion beam, and the detector was positioned at a scattering angle of 91° with an exit angle of 82° (see inset). The sample was oriented such that the scatter plane is perpendicularly aligned to the oxide lines. The titanium signal is found at around 1.15 MeV and the Ru signal at around 1.35 MeV. The higher energies compared to the measurements in Fig. 2a result from the smaller scattering angle. Our studies have also shown that the silicon signal originating from the oxide lines appears at 1.15 MeV, where it overlaps with the titanium signal. Most interestingly, the ruthenium signal appears as a double peak in the glancing exit geometry. It will be shown below that the observed peak shape contains detailed information about the site-specific areal density of ruthenium.

**Figure 3:**
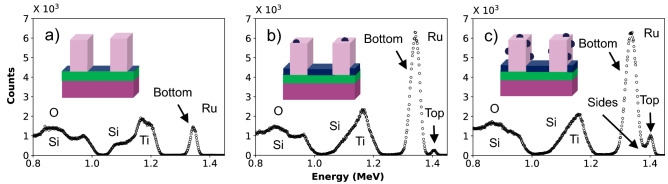
Experimental grazing angle spectra in the same geometry as the spectrum in Fig. 2b of (**a**) the nanostructures without pre-treatment after 50 s of Ru CVD, (**b**) the DMA-TMS pre-treated nanostructures followed by 300 s of Ru CVD, and (**c**) the nanostructures without pre-treatment followed by 400 s of Ru CVD.

By comparing the glancing-exit geometry RBS spectra for the three selected samples we can acquire insight into the effect on the RBS spectra due to the presence of Ru on the different surfaces. This is realized in Fig. 3. A comparison with the micrographs in Fig. 1 and the related sample properties, indicates that the Ru signal shows a double peak when there is deposition on the TiN surface area as well as on the SiO_2_ lines. On the other hand, the Ru signal shows a single peak when there is deposition only on the TiN surface area. This may be qualitatively understood as follows: helium ions that have backscattered from ruthenium at the top of the oxide lines do not experience any energy loss on their way to the detector and appear at 1.4 MeV, whereas helium ions that have backscattered from ruthenium at the bottom of the trench must traverse several oxide lines before reaching to the detector and thus experience a certain energy loss. One may thus expect that Rutherford backscattering allows to determine the relative amount of Ru on the top and bottom of the nanostructures if this energy loss is large enough to disentangle the respective contributions to the backscattering spectrum. Helium ions that have backscattered from the ruthenium on the sidewall will also lose energy in the oxide lines as they travel to the detector, though less as compared to the ones backscattered from ruthenium on the bottom. Depending on whether the Ru was located near the top or near the bottom of the line, the energy loss will be different as the number of traversed lines will be different. Overall, the sidewall signal is expected to emerge at an energy in-between that of the top and bottom signal. Due to the limited energy loss for these cases, the signals related to the various Ru locations do overlap in the final spectrum and a quantitative analysis can only be achieved by modelling the RBS experiment based on the complete 3D elemental distribution.

## Data analysis and interpretation

To model the RBS experiment we need to make use of a simulator. The Rutherford backscattering spectra on the nanostructures were analyzed with STRUCTNRA^27^. The STRUCTNRA software can simulate RBS spectra on patterned samples by simulating multiple pseudo-random beam incidences over a voxelated model of the sample using periodic boundary conditions. The voxel size used in the present work was 0.5 nm × 0.5 nm. The energy loss per voxel is below 1 keV, which is far below the detector resolution of 17.5 keV such that artifacts due to the voxelated sample model are avoided. We verified that a simulation with 100 random incidences sufficiently limits the variance of the simulated spectra in the present case. We used the Ziegler-Biersack stopping power as implemented in the STRUCTNRA code for all the elements except Ru. The stopping power of Ru was increased by 8% to better fit the experimental spectra (see the supplementary material for more information). Further, the ruthenium signal is treated as being superimposed on a very low intensity background attributed to pile-up^28^. The background below the ruthenium signal was estimated based on the intensities that are observed in the regions of the spectra where no signals are expected.

**Figure 4:**
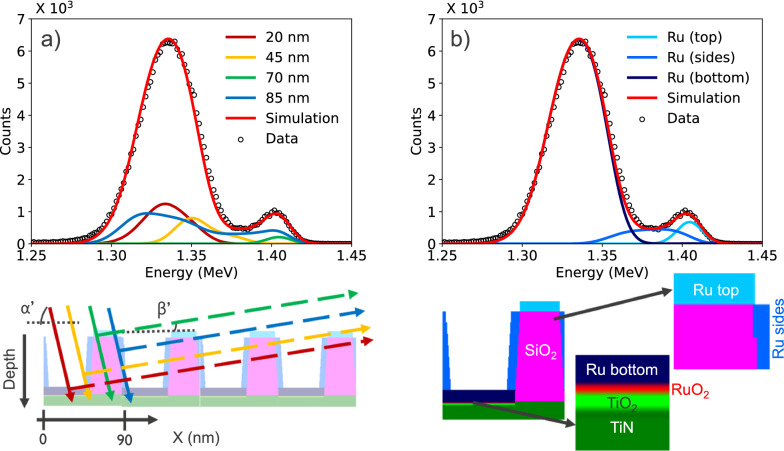
(**a**) Zoom-in on the ruthenium signal for the RBS spectrum on the nanostructures of Fig. 1c. Four simulated sub-spectra that contribute to the summed spectrum are plotted in different colors—their trajectories are drawn on the sample model with the same colors. The labels correspond to the horizontal starting point of the trajectories within STRUCTNRA. (**b**) The Ru contributions originating from different sites (bottom trenches, oxide sidewalls and oxide top surfaces) are plotted.

Besides a simulator, to model the RBS experiment we need a geometrical model of the nanostructures to perform the trajectory simulations. Detailed information about the shape and the dimensions of the nanostructures was obtained from TEM measurements (details in supplementary material). The TEM characterizations were used to construct a voxelated sample model as shown in Fig. 4. The presence of ruthenium on the various surfaces is modelled as a thin layer. This deviates from the observations in the TEM images, which show that the ruthenium on the oxide lines conglomerates in the form of nanoparticles. However, the inclusion of nanoparticles in the sample description in STRUCTNRA would lead to an excessive computational demand due to problems with the periodic boundary conditions. Besides, it would also require detailed information on the size distribution and location of the ruthenium clusters, which at present cannot be derived from the TEM analysis. We have verified that the representation of the nanoparticles by an average layer (with the same overall number of Ru atoms) has no major effect on the simulated spectra when the average size of the nanoparticles is below 10 nm corresponding to an energy loss of below 5 keV. Yet, the presence of larger ruthenium clusters (> 10 nm) may lead to an additional energy broadening in the Rutherford backscattering spectrum, which would not be picked up in our simulations. Similarly, we have verified that a *random* variation of up to 7 nm in the width or the height of the SiO_2_ lines would not significantly affect the quantitative results for the site-specific areal densities.

The methodology in STRUCTNRA of constructing a summed spectrum from multiple sub-spectra is illustrated in Fig. 4a. The figure zooms in on the ruthenium signal and visualizes four sub-spectra corresponding to He ions that impinge on different lateral positions relative to the lines. Note that for each incoming trajectory, the apparent depth (see diagram underneath Fig. 4a) of the scattering event varies, and that the outgoing paths differ in terms of the number of lines traversed before reaching the detector. As expected, the Ru signal from the trajectory solely crossing the top of the line (green color) is found at high energy, the one from the incoming trajectory solely crossing the bottom (dark red color) at low energy. Consequently, the simulation or the fitting of RBS spectra on nanostructures is computationally a hundred times more demanding than for RBS spectra on blanket samples.

Although the voxelated sample model is described by only a limited number of adjustable parameters, we have experienced that the simulations are very sensitive to the dimensions and mass densities of the nanostructures and to the site-specific atom areal densities of ruthenium. Therefore, these sample parameters can be obtained by optimizing the parameters of the voxelated sample model to fit the experimental RBS spectra. The density used for SiO_2_ is 1.90 (10) g/cm^3^ and for TiN is 4.8 (3) g/cm^3^. The refined exit angle of 82.8 (2)° is in good agreement with the nominal value of 82.0 (7)°. Above all, the ruthenium signal in the RBS spectra is *very* sensitive to the site-specific areal densities of Ru in the trenches, on top, and on the sidewalls of the lines. The contributions of ruthenium in the trenches and on the different surfaces of the lines are shown as separate contributions in Fig. 4b.

We are now able to determine the amount of ruthenium on the various regions of the sample, i.e., bottom trenches, sidewall and top of the oxide lines by fitting the RBS spectra (see the supplementary material for details). Nevertheless, given the non-planar nature of the sample, the conventional interpretation of the RBS spectra in terms of an overall areal density is no longer applicable. Indeed, one needs to consider that the Ru is only present on certain areas and thus needs to convert the overall areal density i.e., the apparent amount of ruthenium at the top/sides/bottom per unit area of *substrate*, into a *local areal density* whereby the latter denotes the areal density of ruthenium per unit surface that was exposed to the area-selective deposition. The overall Ru areal density of the top/sides/bottom can thus be converted to the corresponding local Ru areal density by scaling with the substrate area to respective surface area ratio.

**Figure 5:**
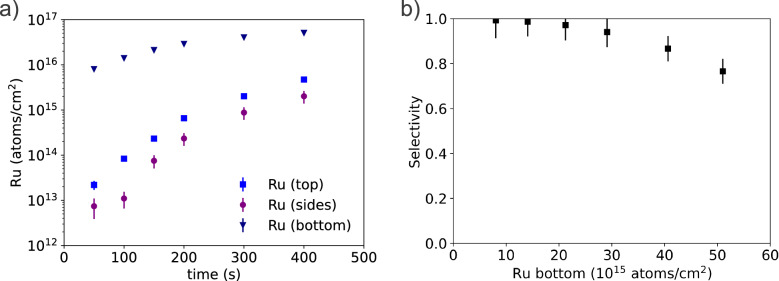
Local areal density (**a**) of Ru in the trench, on the sidewall and on the top of the lines, as a function of Ru CVD deposition time and selectivity (**b**) in function of the Ru areal density at the bottom. If the error bars are not visible, then they are smaller than the data points. For bulk Ru material an areal density of 10^15^ atoms/cm^2^ corresponds to a thickness of 0.14 nm.

In Fig. 5a, we plot the local areal densities for the TiN/SiO_2_ line-space pattern samples without pre-treatment after different times of CVD deposition. At short deposition times (0–100 s), the amount of Ru deposited on the TiN surface area is 300 times higher than that on the top of the SiO_2_ lines, and 1000 times higher than that on the SiO_2_ sidewalls. The observed selectivity, leading to a longer growth delay on the SiO_2_ sidewalls versus top surface, is explained by diffusion of Ru adspecies from the SiO_2_ sidewalls towards the TiN area where aggregation occurs, consistent with the observation made in reference^7^. It is also important to note that by virtue of the ensemble approach, we demonstrate that RBS can quantify local areal densities of ruthenium down to 1.1 (0.4) 10^13^ atoms/cm^2^ on the sidewall after 100 s of deposition, or about 1/100 of a monolayer which is equivalent to a few hundred ruthenium atoms on a line on a standard TEM lamella. Further decrease in the local ruthenium areal density can no longer be distinguished in the current analysis due to the increase of the corresponding relative uncertainty. The reported sensitivity is in line with earlier studies on the sensitivity of RBS^19^. In Fig. 5b we plot the selectivity of the deposition as calculated from the local areal density values in Fig. 5a^3^. A table and linear plot of the data in Fig. 5a can be found in the supplementary material.

The uncertainties in Fig. 5 are estimated by propagating the uncertainties from the counting statistics of the signals, the uncertainty of the stopping power of Si (3%) used to estimate the solid angle ⋅ charge product, the experimental errors on the sample tilt angle and the scattering angle, as well as the uncertainty on the nanostructure dimensions^19,26^. In addition, we have followed three approaches to estimate the uncertainty resulting from the signal deconvolution (see the supplementary material for details). In the first approach, the characterization, including repeating the measurements and the data analysis, was repeated 5 times for a sample with a high local areal density of Ru on all sites of the structures. The sample was moved in between the successive characterizations to avoid beam damage effects and to include local sample homogeneities. We obtain a one-sigma repeatability of 0.5% for Ru at the bottom of the trench, 7% for the Ru at the sides and 4% for the Ru on the top of the oxide lines. In the second approach, we estimated the impact of the scattering geometry error on the deconvolution of the three ruthenium contributions for a similar sample. The measurements are repeated 5 times with varying the exit angle between 83° and 84° in steps of 0.25°. Each spectrum is analyzed by assuming the exit angle to be 83.5°. As a result, the experimental error on the exit angle of 0.7° leads to an uncertainty of 2% for the ruthenium on top, an uncertainty of 30% for the ruthenium on the sides, and of 2% for the ruthenium in the trenches. In the third approach, the impact of the ambiguity of the fit was estimated by varying the local areal density of ruthenium in STRUCTNRA allowing for an increase in the χ^2^ of 5% for the three different contributions around the best fit values. The root of the sum of squares of the uncertainties from the three approaches is used as an estimate for the signal deconvolution uncertainty.

Since the uncertainties are not easily discernable in Fig. 5, they are reported for the two extreme cases. For the sample after 400 s of deposition, the local areal density of Ru at the bottom is 51 (2) 10^15^ atoms/cm^2^, at the sides is 2.0 (0.6) 10^15^ atoms/cm^2^, and at the top is 4.7 (0.3) 10^15^ atoms/cm^2^. For the sample after 100 s of deposition, the local areal density of Ru at the bottom is 14.1 (0.7) 10^15^ atoms/cm^2^, at the sides is 1.1 (0.4) 10^13^ atoms/cm^2^, and at the top is 8.4 (1.1) 10^13^ atoms/cm^2^. Overall, we have found that the uncertainty due the deconvolution dominates for large local areal densities, while the uncertainties due to the counting statistics and the background signal dominate for samples with a low local areal density.

This approach is a promising analytical method for studying the mechanism of selective deposition and selectivity loss during area-selective deposition in nanoscale structures, where the behavior can be different as compared to large scale structures^7^. In addition, one could question whether the followed approach is generally applicable to study the area-selective deposition in periodic nanostructures but with different geometrical dimensions. To estimate whether the Ru signal from the bottom and from the top of the lines can be differentiated, one needs to estimate the path length of the ions in the SiO_2_ lines and require that the energy loss must be larger than the detector energy resolution. For the method to be applicable, the height of the considered nanostructures can be reduced to 25 nm, the pitch can be increased up to 205 nm, or the width of the lines can be reduced to 15 nm. Generally, a smaller pitch allows for the analysis of smaller nanostructures.

Finally, we report on the possibility to improve the lateral or vertical resolution, for example to quantify the ruthenium local areal density on the side walls as a function of distance from the bottom of the trenches. We explored the increase of the exit angle (82° to 84°) to increase the path length of the backscattered ions in the lines. We also explored the decrease of the primary ion beam energy to 1.0 MeV to increase the stopping power^19^. However, we found that both approaches also amplify the effect of the non-uniformity of the nanoparticles resulting in a Ru signal which cannot be modeled anymore as a uniform average coverage. Whereas the present experimental conditions work well for average cluster sizes up to 10 nm, it is expected that the mentioned optimizations may become beneficial when the average cluster sizes are smaller.

## Conclusion

We showed that site-specific compositional information from periodic nanostructures can be obtained by using Rutherford backscattering spectrometry. The enhanced energy loss effect in a grazing exit geometry is used to obtain the site-specific information. An essential aspect in the approach is that an ensemble of identical nanostructures is probed, leading to a high sensitivity and fast analysis times. The approach is demonstrated for Ru area-selective deposition on SiO_2_-TiN line-space patterns with a width of 35 nm, where the Ru growth evolution on the different areas as well as the selectivity of the deposition is quantified with a limit of detection of 10^13^ atoms/cm^2^. The high sensitivity of RBS and the capability to provide absolute quantification of the selectivity make this an attractive analytical method to investigate the fundamentals of area-selective deposition in advanced nanostructures for the semiconductor technology.

## Supplementary Information

Supplementary Information.

## Data Availability

The data used for this study can be obtained from the corresponding author on reasonable request.
