# Supplementary material for: Quantification of area-selective deposition on nanometer-scale patterns using Rutherford backscattering spectrometry
Source: Sci Rep. 2022 Oct 22;12:17770. doi: 10.1038/s41598-022-22645-8 (PMC9587986; doi:10.1038/s41598-022-22645-8)
Supplement: Supplementary file 1 — Supplementary Information. [file 41598_2022_22645_MOESM1_ESM.pdf]

Quantification of area-selective deposition on nanometer-scale patterns using Rutherford backscattering spectrometry (supplementary material)

Niels Claessens[1,2], Zamran Zahoor Khan[1], Negin Rahnemai Haghighi[1,3], Annelies Delabie[1,3], André Vantomme[2], Wilfried Vandervorst[1,2], Johan Meersschaut[1].

[1] IMEC, Kapeldreef 75, 3001 Leuven, Belgium
[2] Quantum Solid State Physics, KU Leuven, Celestijnenlaan 200D, 3001 Leuven, Belgium
[3] Quantum Chemistry and Physical Chemistry, KU Leuven, Celestijnenlaan 200F, 3001 Leuven, Belgium

[*] Corresponding Author (niels.claessens@imec.be)

confidential

S1. Simulation parameter settings and optimization

The Rutherford backscattering spectra on the nanostructures in the main text are analyzed using STRUCTNRA [1]. Initially, we used the Ziegler-Biersack stopping power as implemented in the STRUCTNRA code for all the elements but noticed that the simulated Ru signals were poorly fitting the experimental data. To test the correctness of the simulation parameters and exclude effects of the Ru clusters we investigated the spectra of a conformal Ru/TiN/SiO_2/Si blanket film sample using SIMNRA simulations [2]. Naturally, SIMNRA and STRUCTNRA share the same stopping power tables and energy loss implementation.

As can be seen in figure S1-a. the Ru signal is much sharper simulated than the experimental data suggests for the blanket film. There are two plausible explanations for this phenomenon being surface roughness or underestimation on the Ru stopping power in the used stopping data sets. Given that we expect the surface roughness to be small and given the fact that the required roughness for a better fit is of the order of the layer thickness, we conclude that the Ru stopping power must be underestimated. We found that an increase of 8% on the Ru stopping power was necessary to properly fit the experimental data as can be seen in figure S1-b. Unfortunately, to our knowledge, the stopping power of Ru remains experimentally undetermined. Given that the experimental stopping powers of similar elements differ up to 10% around 1.5 MeV, deviations of this magnitude are to be expected [3].

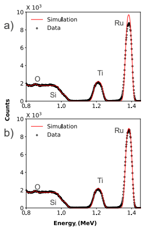

Figure S1 (a) Simulation of the blanket film spectrum in grazing angle geometry using the standard stopping tables of SIMNRA [2]. (b) Simulation generated with the same simulation parameters except for the stopping power of Ru which is increased by 8%.

Since the STRUCTNRA software is based on the simulation of pseudo-random beam incidences to average over a voxelated model of the sample, the number of random incidences needs to be optimized considering the gain in precision, and simulation time [1]. In figure S2, for the case of the nanostructure without pre-treatment followed by 400 s of Ru CVD, we plot the reduced χ^2 between the simulated spectrum after 1000 incoming trajectories and the simulation with a limited number of incoming trajectories. The uncertainty is estimated by calculating the standard deviation on the reduced χ^2 for five simulations. For the present case we estimate that a set of 100 random incoming trajectories is sufficient to simulate the spectra.

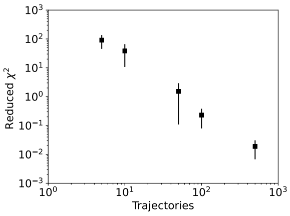

Figure S2 Reduced χ^2 of the simulated RBS spectra of the nanostructure without pre-treatment followed by 400 s of Ru CVD in grazing angle geometry in function of the number of incoming trajectories as compared to a simulation of 1000 incoming trajectories. The simulations were performed using STRUCTNRA [1].

S2. TEM analysis of the geometrical sample parameters

To limit the number of fitting parameters we acquire geometrical information on the nanostructures from cross-TEM images. These images supply us with the width and height of the features of the nanostructures. The dimensions of 10 lines are used to estimate the average dimension of the nanostructures. The uncertainty on the dimensions is estimated by calculating the standard deviation. The results for the nanostructures shown in figure 1 (main text) are plotted in figure S3.

confidential

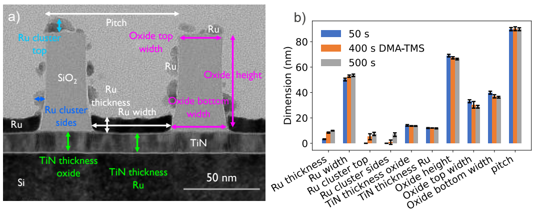

Figure S3 (a) TEM image of the nanostructures without pre-treatment after 400 s of Ru CVD with annotated dimensions. (b) Average dimension of the most prominent features of the nanostructures without pre-treatment after 50 s of Ru CVD (50 s), the DMA-TMS treated nanostructures after 300 s of Ru CVD (400 s DMA-TMS), and the nanostructures without pre-treatment after 400 s of Ru CVD (500 s) as determined by a TEM analysis.

S3. Fitting of the 3D Ru distribution

First, we refined the SiO_2 density, the TiN density and experimental geometry (scattering angle, tilt angle). Our procedure to assess these is as follows.

1. The density of TiN was fixed using the average Ti areal densities estimated from the RBS spectra taken at 170° scattering angle assuming stoichiometric TiN and the physical thicknesses estimated from TEM. The found TiN densities average to 4.8(3) g/cm^3 and were equal within the uncertainty with the nominal value of 4.85(15) g/cm^3.
2. The density of SiO_2 and experimental geometry were manually optimized such that the induced energy loss on the signal from Ru on the bottom of the trench agrees well with the experimental data while ignoring the eventual presence of Ru on the top/sidewall. This is justified since the energy loss due to the Ru at the top and sides is found to be negligible compared to that of the SiO_2 lines (3 keV to 40 keV). To assess the energy loss of the Ru density at the bottom of the trench, the experimental Ru double peak structure was split in two peaks whereby the Ru signal at high energy was assigned to Ru at the top and low energy peak to Ru at the bottom. To estimate the uncertainty on the results related to the density of SiO_2 and experimental geometry, we performed a sensitivity analysis by varying these slightly. However, since most of the energy is lost in the outgoing path, determined by the exit angle, the effect on the simulated spectra of changing the tilt or the scattering angle is virtually identical. Therefore, only the scattering angle was varied while keeping the tilt angle constant. The found SiO_2 densities and exit angles are similar and average to 1.90(10) g/cm^3 and 82.8 (2)° in good agreement with the nominal value of 2.00(10) g/cm^3 and 82.0 (7)°.

Second, we estimated the local areal densities of ruthenium. Our procedure to estimate these is as follows.

1. The charge solid angle product is estimated using the simulated/observed number of Si counts from the oxide lines and Ti counts.
2. As discussed in the main text, the counts of the different Ru signals originating from the top, sidewall, and bottom of the structure give a contribution in three different energy ranges. For example, in the case of the nanostructures without pre-treatment after 400 s of Ru CVD these ranges are taken to be 1.270 MeV – 1.375 MeV for the bottom, 1.375 MeV – 1.390 MeV for the sidewall and 1.390 MeV – 1.440 MeV for the top. The local areal densities of ruthenium are manually optimized such that the simulated number of counts in these three energy ranges of the Ru sub-spectrum are equal to the observed number of counts within these ranges. This step is introduced to limit the number of simulations necessary for a good fit.
3. After the manual optimization using the total number of counts in each region, an automated fitting of the local areal densities of Ru based on the minimization of the reduced χ^2 between the simulation and experimental spectra was performed as discussed below. Generally, the manual optimization already yields a very good fit to the data such that the automated fit only changes the areal density values slightly. The average areal density of ruthenium estimated from the RBS spectra taken at 170° scattering angle and the one estimated from the grazing angle measurement by rescaling and summing the three local areal densities are found to be equal within the uncertainty of the experiment.

As discussed above we varied the local areal densities of Ru automatically after a manual optimization to fit the Ru part of the spectra. Figure S4 shows the effect of varying the local areal density of ruthenium at the bottom by +-50% for the simulated spectra of the nanostructures without pre-treatment after 400 s of Ru CVD. Figure S4-a. shows the direct effect on the spectrum. To increase the sensitivity of the reduced χ^2 to the different local areal densities we evaluate the reduced χ^2 in the energy range of the corresponding signal as indicated in Figure S4-a. (1 = bottom, 2 = sides, and 3 = top). Figure S4-b to S4-d plot the reduced χ^2 in these energy ranges in function of the local areal density of Ru at the bottom. We repeated this analysis for the three local areal densities. The slopes away from the minimum of the reduced χ^2 in the different regions in function of the local top, sides and bottom areal densities around the minimum are shown in table S1. It is clear from these values that the changing the bottom local areal density of reduced χ^2 in region 2 is nearly independent on the bottom, and top local areal

density of ruthenium. The reduced χ^2 in region 1 is less dependent on the sides, and top local areal density of Ru compared to the χ^2 in region 3 on the bottom and sides local areal density of Ru. We thus opted to first fit the sides, then the top, and finally the bottom areal density to minimize the effects of correlation between the local areal densities.

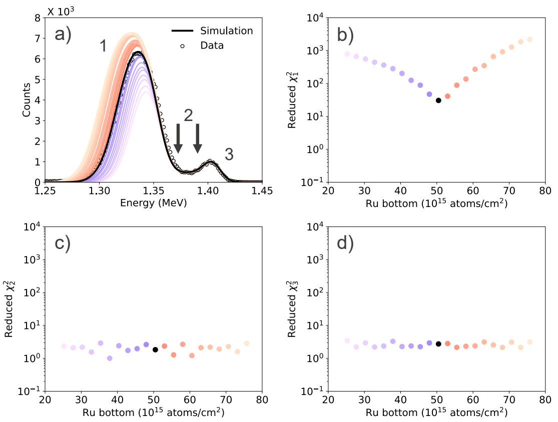

Figure S4 Effects of the variation of the bottom local areal density of Ru for the simulated spectra in grazing angle geometry for the nanostructures without pre-treatment after 400 s of Ru CVD. (a) The experimental and simulated spectra for different local areal densities of ruthenium at the bottom. The arrows indicate the regions in which the reduced χ^2 is evaluated. (b-d). Reduced χ^2 in the indicated regions as a function of the bottom areal density of Ru.

	$\frac{\Delta\chi_1^2}{\Delta x}$	$\frac{\Delta\chi_2^2}{\Delta x}$	$\frac{\Delta\chi_3^2}{\Delta x}$
Ru bottom	58	0.010	0.0081
Ru sides	13	96	16
Ru top	2.6	0.92	16

Table S1 Estimated average local slope in the minimum of the reduced χ^2 evaluated in the different regions in function of the Ru bottom, sides, and top local areal densities for the nanostructures without pre-treatment after 400 s of Ru CVD.

The characterization, including repeating the measurements and the data analysis, is repeated 5 times for a sample with a high local areal density of Ru on all sites of the structures. We plot the resulting spectra in figure S5-a and the corresponding local areal densities of the top, sides, and bottom in figure S5-b together with the calculated standard deviation which is shown in table S2. To estimate the impact of errors on the exit angle we repeat the grazing angle experiments for a similar sample while varying the exit angle between 83° and 84° in steps of 0.25°. We plot the resulting spectra in figure S6-a and the resulting local areal densities of the top, sides, and bottom Ru in figure S6-b. The found values follow a linear trend with a slope indicated in table S3. The slope can be thought of as an estimate for the sensitivity to errors made on the exit angle.

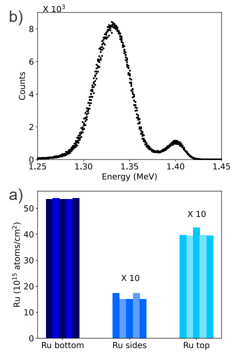

Figure S5 (a) Several identical measurements of the sample in grazing angle geometry. (b) The found local areal density of Ru at the top, sides, and bottom sites.

	Repeatability (%)
Ru top	4
Ru sides	7
Ru bottom	0.5

Table S2 Repeatability of the local areal density of Ru.

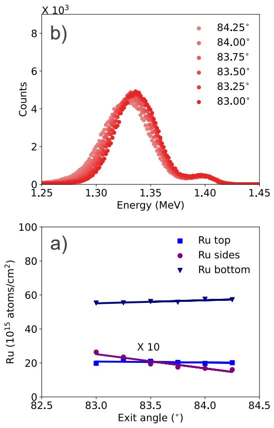

Figure S6 (a) Several measurements at varying exit angle of the sample in grazing angle geometry. (b) The found local areal density of Ru at the top, sides, and bottom as a function of the exit angle. The error bars are smaller than the data dots. A linear trendline is added.

	Slope (%/°)
Ru top	-3
Ru sides	-40

Ru bottom	3

Table S3 Estimated slope of the local areal density in function of the exit angle. The slope is normalised to the average of the data points.

S4. Information relevant to area-selective deposition

In the main text the local areal densities for the TiN/SiO_2 line-space pattern samples without pre-treatment after different times of CVD deposition is plotted in log-scale in figure 5-a. In the area-selective deposition communities usually this data is presented in a linear-scale. For completion we plot the same data as in figure 5 of the main text with both graphs in linear scale in figure S7. We also tabulate the data from figure 5-a in table S4.

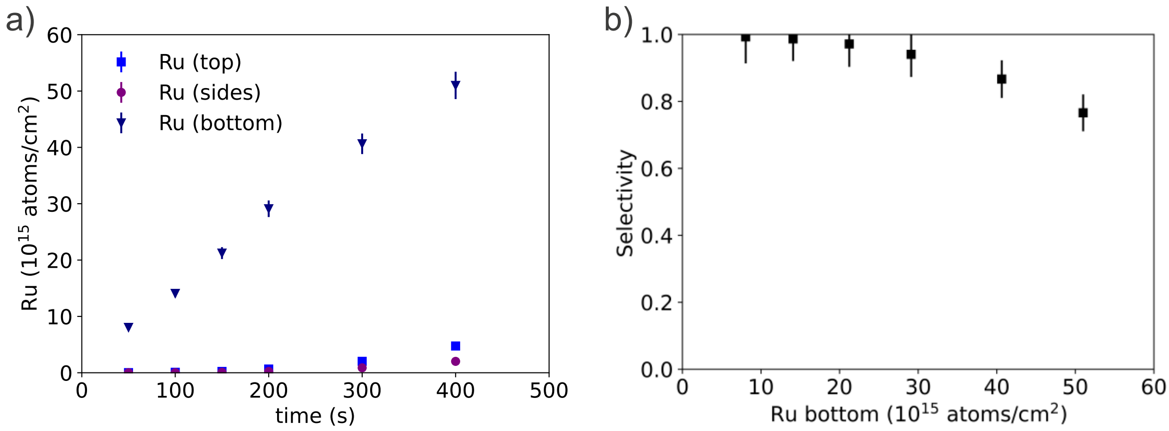

Figure S6 Local areal density (a) of Ru in the trench, on the sidewall and on the top of the lines, as a function of Ru CVD deposition time and selectivity (b) in function of the Ru areal density at the bottom. If the error bars are not visible, then they are smaller than the data points. For bulk Ru material an areal density of 10^{15} atoms/cm² corresponds to a thickness of 0.14 nm.

time (s)	Ru (top)	uncertainty	Ru (sides)	uncertainty	Ru (bottom)	uncertainty
**50**	0.022	0.005	0.007	0.004	8.0	0.5
**100**	0.084	0.011	0.011	0.004	14.1	0.7
**150**	0.23	0.02	0.08	0.02	21.2	1.1
**200**	0.66	0.05	0.23	0.07	29.1	1.5
**300**	2.03	0.15	0.9	0.3	40.6	1.8
**400**	4.7	0.3	2.0	0.6	51	2

Table S4 Local areal density in 10^{15} atoms/cm² of Ru in the trench, on the sidewall and on the top of the lines, as a function of Ru CVD deposition time.

References

[1] Mayer, M. Computer simulation of ion beam analysis of laterally inhomogeneous materials. *Nucl. Instrum. Methods Phys. Res., B*. **371**, 90-96 (2016).

[2] Mayer, M. SIMNRA, a Simulation Program for the Analysis of NRA, RBS and ERDA. *Nucl. Instrum. AIP Conf. Proc*. **475**, 541-544 (1999).

[3] Paul, H. Stopping Power for Light Ions: Graphs, Data, Comments and Programs http://www-nds.iaea.org/stopping/.

Legends

Figure S1 (a) Simulation of the blanket film spectrum in grazing angle geometry using the standard stopping tables of SIMNRA [2]. (b) Simulation generated with the same simulation parameters except for the stopping power of Ru which is increased by 8%.

Figure S2 Reduced χ^2 of the simulated RBS spectra of the nanostructure without pre-treatment followed by 400 s of Ru CVD in grazing angle geometry in function of the number of incoming trajectories as compared to a simulation of 1000 incoming trajectories. The simulations were performed using STRUCTNRA [1].

Figure S3 (a) TEM image of the nanostructures without pre-treatment after 400 s of Ru CVD with annotated dimensions. (b) Average dimension of the most prominent features of the nanostructures without pre-treatment after 50 s of Ru CVD (50 s), the DMA-TMS treated nanostructures after 300 s of Ru CVD (400 s DMA-TMS), and the nanostructures without pre-treatment after 400 s of Ru CVD (500 s) as determined by a TEM analysis.

Figure S4 Effects of the variation of the bottom local areal density of Ru for the simulated spectra in grazing angle geometry of the nanostructures without pre-treatment after 400 s of Ru CVD. (a) The experimental and simulated spectra for different local areal densities of ruthenium at the bottom. The arrows indicate the regions in which the reduced χ2 is evaluated. (b-d). Reduced χ2 in the indicated regions as a function of the bottom areal density of Ru.

Figure S5 (a) Several identical measurements of the sample in grazing angle geometry. (b) The found local areal density of Ru at the top, sides, and bottom sites.

Figure S6 (a) Several measurements at varying exit angle of the sample in grazing angle geometry. (b) The found local areal density of Ru at the top, sides, and bottom as a function of the exit angle. The error bars are smaller than the data dots. A linear trendline is added.

Table S1 Estimated average local slope in the minimum of the reduced $\chi 2$ evaluated in the different regions in function of the Ru bottom, sides, and top local areal densities for the nanostructures without pre-treatment after 400 s of Ru CVD.

Table S2 Repeatability of the local areal density of Ru.

Table S3 Estimated slope of the local areal density in function of the exit angle. The slope is normalised to the average of the data points.

Figure S6 Local areal density (a) of Ru in the trench, on the sidewall and on the top of the lines, as a function of Ru CVD deposition time and selectivity (b) in function of the Ru areal density at the bottom. If the error bars are not visible, then they are smaller than the data points. For bulk Ru material an areal density of 1015 atoms/cm² corresponds to a thickness of 0.14 nm.

Table S4 Local areal density in 10^{15} atoms/cm² of Ru in the trench, on the sidewall and on the top of the lines, as a function of Ru CVD deposition time.

confidential
